# Dichloroacetate Overcomes Oxaliplatin Chemoresistance in Colorectal Cancer through the miR-543/PTEN/Akt/mTOR Pathway

**DOI:** 10.7150/jca.34650

**Published:** 2019-10-15

**Authors:** Yu Liang, Danxi Zhu, Liming Zhu, Yichao Hou, Lidan Hou, Xin Huang, Linjing Li, Yu Wang, Lei Li, Huimin Zou, Tianqi Wu, Mengfei Yao, Jianhua Wang, Xiangjun Meng

**Affiliations:** 1 Department of Gastroenterology, Shanghai Ninth People's Hospital, Shanghai Jiao Tong University School of Medicine, Shanghai, China.; 2 Cancer institute, Fudan University Shanghai Cancer Center, Fudan University, Shanghai, China.

**Keywords:** colorectal cancer, oxaliplatin, chemoresistance, miR-543 colorectal cancer, oxaliplatin, chemoresistance, miR-543

## Abstract

Chemoresistance is responsible for most colorectal cancer (CRC) related deaths. In this study, we found that dichloroacetate (DCA), a pyruvate dehydrogenase kinase (PDK) inhibitor, can be used as a sensitizer for oxaliplatin (L-OHP) chemoresistant CRC cells. The aim of this study was to explore the ability of DCA to overcome L-OHP resistance in CRC cells and to identify the underlying molecular mechanisms. We found that DCA sensitizes chemoresistant CRC cells to L-OHP-induced cytotoxic effects by inhibiting clone formation capacity and promoting cell apoptosis. A microRNA (miRNA) array was used for screen, and miR-543 was identified and shown to be downregulated after DCA treatment. The expression of miR-543 was higher in chemoresistant CRC cells than in chemosensitive CRC cells. Overexpression of miR-543 increased chemoresistance in CRC cells. The validated target gene, PTEN, was negatively regulated by miR-543 both *in vitro* and *in vivo*, and PTEN was upregulated by DCA through miR-543. In addition, overexpression of miR-543 reversed the inhibition of colony formation after DCA treatment. Furthermore, the Akt/mTOR pathway is activated by miR-543 and is involved in the miR-543 induced chemoresistance. There was a significant inverse relationship between miR-543 expression and PTEN level in CRC patients, and high miR-543 expression was associated with worse prognosis. In conclusion, DCA restored chemosensitivity through miR-543/PTEN/Akt/mTOR pathway, and miR-543 may be a potential marker or therapeutic target for chemoresistance in CRC.

## Introduction

Colorectal cancer (CRC) is a major cause of cancer-related mortality in the United States and China 1-3. The present treatment for CRC comprises surgery, chemotherapy, and radiation 4. Although chemotherapy represents one of the major strategies, especially for advanced stage and metastatic CRC, the development of resistance poses a significant limitation to the efficacy of the clinical response to chemotherapy 5. Hence, elucidating the mechanisms of chemoresistance is critical for CRC treatment.

Recently, immunotherapy and biotherapeutic agents have been shown to have an encouraging future in cancer treatment, but drug resistance and inevitable adverse effects constitute limitations clinically. To date, chemotherapy is still a common choice, especially for patients with unresectable late stage cancer^6^, ^7^. Thus, developing novel combinations of classic anticancer drugs and exploring the mechanisms of drug resistance are imperative. As oxaliplatin (L-OHP) is the most commonly used chemotherapeutic drug for CRC, L-OHP-resistant CRC cell lines were used in this study.

Dichloroacetate (DCA), a regulator of metabolism, is used to lower lactic acid levels and treat lactic acidosis, and hereditary mitochondrial diseases have been reported to have an antitumor effect 8, 9. Recently, it has been reported that DCA attenuates hypoxia-induced resistance to 5-fluorouracil in gastric cancer 10, overcomes sorafenib resistance in hepatocellular carcinoma^11^, and reverses cisplatin resistance in head and neck cancer 12. Therefore, DCA may be a sensitizer to chemotherapeutic agents for CRC, especially for chemoresistant CRC. However, the mechanism underlying the effect of DCA on CRC treatment remains elusive.

MicroRNAs (miRNAs) are a class of noncoding RNAs. miRNAs are associated with many biological processes, including cell proliferation, migration, apoptosis, metabolism and chemoresistance 13, 14. Although the functions of numerous miRNAs have been determined, there are still many miRNAs whose functions are unknown or not fully characterized. The present study aimed to elucidate the mechanism underlying the action of DCA, and a microarray screening assay was used to explore potential miRNAs involved in DCA treatment in CRC cells.

By treating CRC cells with DCA, the present study aimed to elucidate the roles of the related miRNA and its target gene to identify the signaling pathway involved in chemoresistance.

## Materials and methods

### Cancer tissue

The present study included 23 patients who were clearly diagnosed with CRC between 2013 and 2016 at the Ninth People's Hospital affiliated with Shanghai Jiao Tong University School of Medicine. All tissues were obtained after obtaining informed consent, and all procedures involving human patients were conducted in accordance with the regulations set forth by the Ethical Committee of the Ninth People's Hospital affiliated with the Medical College of Shanghai Jiao Tong University. The clinical information of the CRC patients is presented in Table 1.

### Cell culture

The L-OHP-resistant cell line HCT-8/L and its parental cell line HCT-8 were purchased from iCell Bioscience, Inc. (Shanghai, China). The L-OHP-resistant cell line HCT116/L was provided by Dr. Lin from the Cancer Hospital of the Chinese Academy of Medical Sciences. The HCT 116 cell line and the 293T human embryonic kidney 293T cell line were obtained from the American Type Culture Collection (ATCC, Manassas, VA, USA). HCT116 and 293T cell lines were cultured in Dulbecco's Modified Eagle Medium. HCT-8 cells were cultured in RPMI-1640 medium (HyClone, Utah, US). The culture medium was supplemented with 10% foetal bovine serum (Gemini, California, US), 100 U/mL penicillin and 100 μg/mL streptomycin (HyClone). The culture medium of the resistant CRC cells was supplemented with 5 μg/ml L-OHP. All cell lines were authenticated by short tandem repeat (STR) sequencing by Genetic Testing Biotechnology Corporation (Suzhou, China). DCA was purchased from Sigma-Aldrich Co. Ltd. (MO, USA).

### Cell growth assay

Cells were seeded in 96-well plates at a density of 5000 cells/well. After overnight incubation, cells were treated with drugs for 24 hours. Culture medium was then replaced with 10 μl of cell counting kit-8 (CCK8) reagent (Dojindo, Japan) and 90 μl of fresh medium. Absorbance at an optical density (OD) value of 450 nm was measured by an enzyme microplate reader (BioTeck, VT, US) 2 hours later. The survival rate was calculated using the following formula: OD_drug_/ OD_ctrl._ The half maximal inhibitory concentration (IC50) of L-OHP was calculated by GraphPad Prism 6 (GraphPad Software, San Diego, CA).

### Colony formation assay

Cells were seeded in 6-well plates at a concentration of 1000 cells per well. After overnight incubation, drugs were added to the cell suspension. After incubation at 37°C for 1 to 2 weeks, cells were fixed with 4% paraformaldehyde for 30 min and stained with 1% crystal violet. The number of colonies was counted by a counter (Gelcount, Optronix, Oxford).

### miRNA microarray

HCT116 cells were treated with 5mM, 10mM or 20mM DCA for different times. Total RNA was isolated using TRIzol (Invitrogen) and purified with an RNeasy mini kit (QIAGEN) according to the manufacturer's instructions. RNA quality and quantity were measured using a nanodrop spectrophotometer (ND-1000, Nanodrop Technologies) and RNA Integrity was determined by gel electrophoresis performed by Kangchen Biotech (Shanghai, China). RNA labelling and array hybridization were performed according to Exiqon's manual. Scanned images were then imported into GenePix Pro 6.0 software (Axon) for grid alignment and data extraction. Replicated miRNAs were averaged, and miRNAs with intensities ≥30 in all samples were selected for calculating the normalization factor. Expressed data were normalized using the median normalization. After normalization, significant differentially expressed miRNAs between two groups were identified through fold-change and P-value screening, as were differentially expressed miRNAs between two samples.

### Quantitative real-time PCR

Total RNA from tissues or cells was extracted using TRIzol Reagent (Life, CA, USA). - cDNA was synthesized using the PrimeScript RT Reagent Kit (TaKaRa, Tokyo, Japan). Quantitative real-time PCR was performed using Premix Ex Taq 420A (TaKaRa, Tokyo, Japan) on the ABI-7500 platform according to the manufacturers' instructions. All reactions were performed in a 20 μl reaction volume in triplicate. Standard curves were generated and the relative amount of target gene mRNA or miRNA was normalized to actin or U6. The primer sequences are listed in Table A.

### Western blot analysis

Protein lysates (20 μg) were separated by sodium dodecyl sulphate-polyacrylamide gel electrophoresis. Primary antibodies, including cleaved-PARP, Bax, PTEN, p-Akt (Ser473), p-mTOR (Ser2448), mTOR and Akt, were purchased from CST. α-tubulin and GADPH were purchased from Proteintech. The secondary antibodies were purchased from Sungene (Tianjin, China). Proteins were detected using a chemiluminescence imaging system (Bioshine, Shanghai, China).

### Oligonucleotide transfection

miR-543 mimics, inhibitors, and their corresponding controls were synthesized by GenePharma (Shanghai, China). Lipofectamine 3000 (Invitrogen, CA, USA) at a final concentration of 50 nmol/L (mimics and siRNAs) or 100 nmol/L (inhibitors) was used for transfection. The sequences of oligonucleotides are listed in Table B.

### Establishment of stable cell lines

The miR-543 overexpression and control plasmids, as well as the packaging and envelope plasmid were purchased from Yile (Shanghai, China). Lentivirus particles were harvested 48 hours after lentiviral vector transfection into human embryonic kidney 293T cells. Cells were infected with recombinant lentivirus and selected by puromycin. The expression of miR-543 in HCT 116/miR-543-overexpressing and HCT 116/vector cells was confirmed by quantitative real-time PCR.

### Xenograft in nude mice

Six-week-old male nude mice were subcutaneously injected with 7×10^6^ HCT-116/miR-543 overexpressing cells or HCT-116/vector cells. Twenty days after inoculation, the mice were injected intraperitoneally with L-OHP (5 mg/kg) every other day for 3 weeks. Tumor volume was measured every 3 days. Tumor-bearing mice were sacrificed 42 days after inoculation, and the tumors were then dissected, weighed, and frozen at -80°C for further study. All experiments and animal care procedures were approved by the Shanghai Medical Experimental Animal Care Commission.

### Immunohistochemical staining (IHS)

Paraffin-embedded tissues were sliced and incubated with primary antibodies, including Ki67(1;100, Servicebio, Wuhan, China), PTEN (1:100, Servicebio, Wuhan, China) and p-mTOR (1:100, Abcam, Cambridge, UK) at 4°C overnight, and the tissues were then incubated with secondary antibodies. The chromogenic reaction was performed with 3,3-diaminobenzidine (DAB). After counterstaining with haematoxylin, slides were evaluated under a light microscope. The immunoreactive score (IRS) was calculated as follows: IRS= staining intensity (SI) × -percentage of positive cells (PP). SI was categorized as follows: 0 = negative; 1 = weak; 2 = moderate; and 3 = strong. PP was defined as 0 = 0%; 1 = 0-25%; 2 = 25-50%; 3 = 50-75%; and 4 = 75-100%.

### Statistical analysis

Data were analysed by GraphPad Prism 7.0 software. Data are shown as the means ± SD/SEM. All experiments were repeated at least three times. Student's t-test was used to compare differences between the two groups. One-way ANOVA followed by Bonferroni multiple comparisons was used to compare more than two groups. The Kaplan-Meier curves for survival analyses were determined using the log-rank test. The relationship between miR-543 and PTEN was evaluated using spearman's rank correlation coefficient analysis. A *P*-value less than 0.05 was considered statistically significant.

## Results

### DCA acts synergistically with L-OHP in chemoresistant CRC cells

**Figure 1:**
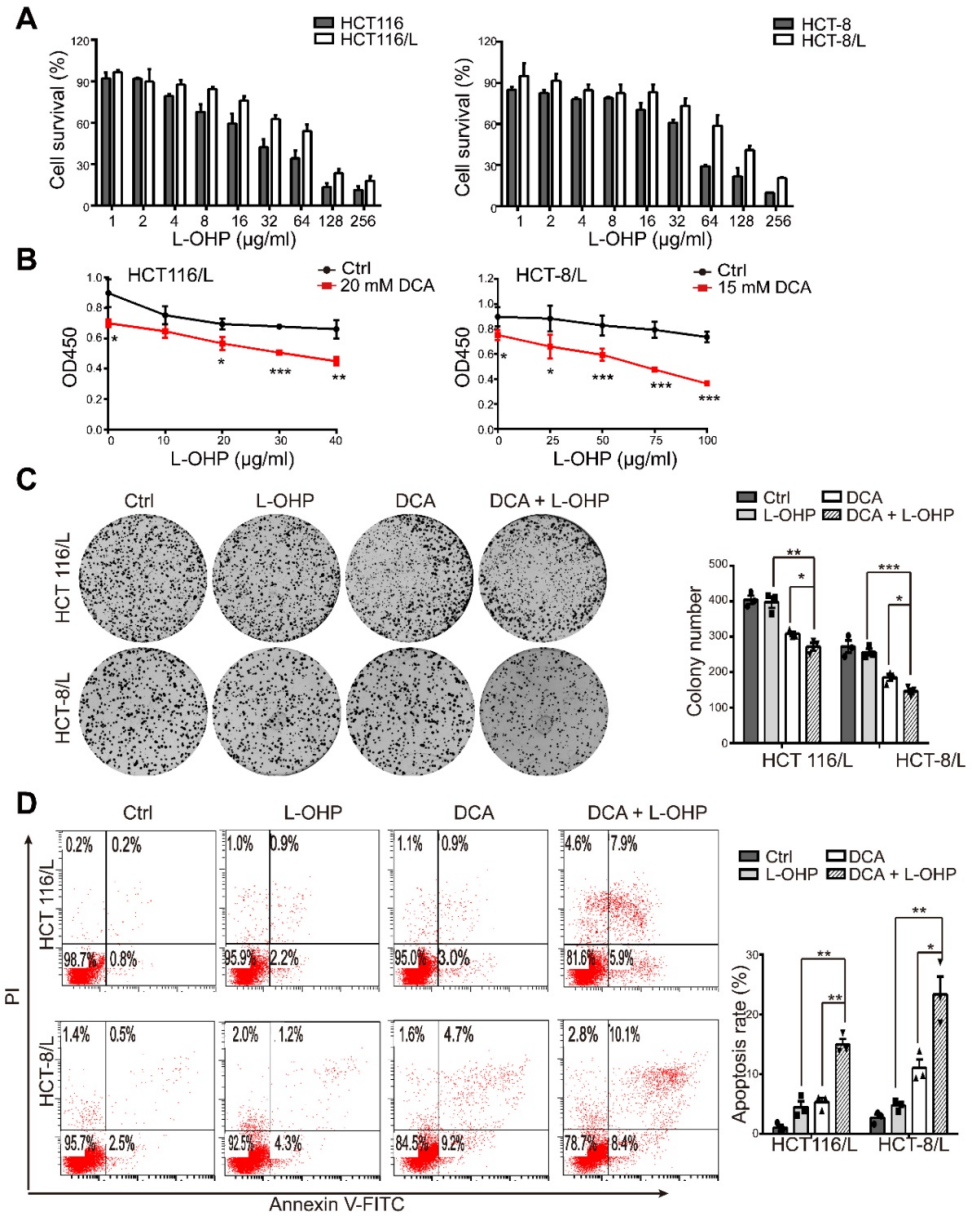
** DCA restores chemosensitivity in L-OHP resistant CRC cells.** (A) HCT116, HCT-8, HCT-116/L, and HCT-8/L cells were treated with different concentrations of L-OHP, and cell survival was measured by CCK8. (B) The OD 450 determined by CCK8 was considered as the cell growth rate. (C-D) HCT116/L and HCT-8/L cells were treated with DCA or/and L-OHP for 24 hours. A colony formation assay was performed, and cell apoptosis was measured. The data shown are representative of three independent experiments. Mean ± SEM are presented, n = 3. *, *P* < .05; **, *P* < .01; ***, *P* < .001.

First, we examined the cytotoxic effect of L-OHP on chemoresistant and chemosensitive CRC cells using CCK8, HCT116/L and HCT-8/L cells were not sensitive to L-OHP compared to the sensitivity of their parental cell lines HCT116 and HCT-8, respectively (Figure 1A). The IC50 values of DCA in HCT116 and HCT-8 cells were approximately 20 and 15mM, respectively, which was in accordance with the findings of previous reports 15, 16. Thus, DCA significantly overcame L-OHP resistance in HCT116/L and HCT-8/L cells, as indicated by cell growth measurements (Figure 1B). Colony formation capacity was also significantly inhibited in the combination treatment group compared to the colony formation capacities of the other groups (Figure 1C). Furthermore, co-treatment with L-OHP and DCA promoted cell apoptosis (Figure 1D). These data suggest that DCA sensitizes CRC cells to L-OHP therapy.

### MiR-543 is regulated by DCA and favoures chemoresistance in CRC cells

**Figure 2:**
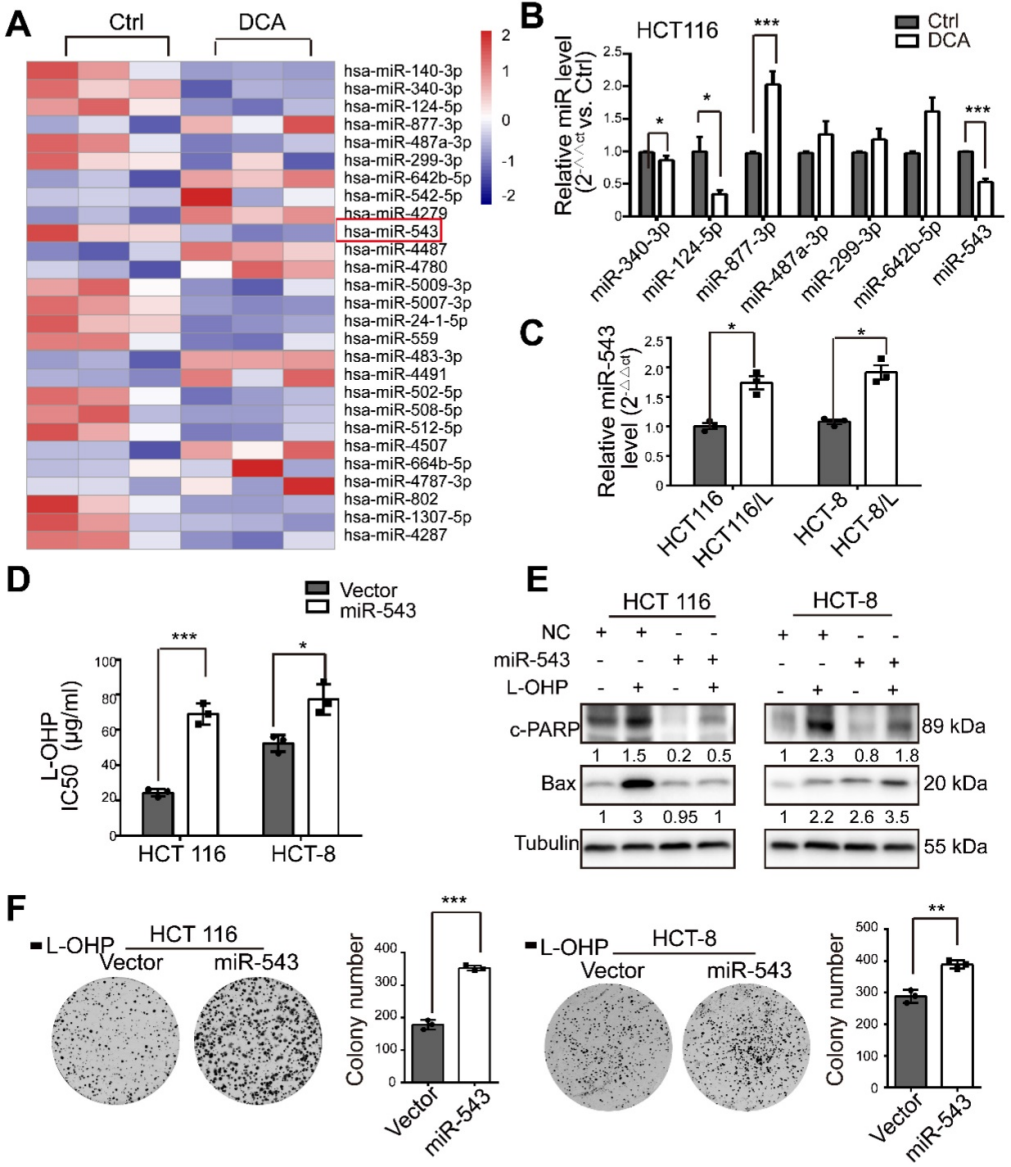
MiR-543 is downregulated after DCA treatment and is involved in chemoresistance. (A) Heatmap of differentially expressed microRNAs in HCT116 cells treated with 20 mM DCA for 24 hours. (B) Total RNA was prepared from HCT116 cells 24 hours after DCA treatment. Multiple microRNAs in microarray data sets were quantified by quantitative real-time PCR. (C) The basal level of miR-543 was determined by quantitative real-time PCR in L-HOP sensitive CRC cells and L-OHP resistant CRC cells. (D-F) HCT116 and HCT-8 cells transfected with miR-543 mimic or infected with overexpression virus were treated with or without L-OHP for 24 hours. The IC50 of L-OHP, the levels of c-PARP and Bax, and colony formation assays were analyzed. The data shown are representative of three independent experiments. Mean ± SEM are presented, n = 3. *, *P* < .05; **, *P* < .01; ***, *P*<.001; ns, no significance.

To comprehensively assess the effects of miRNAs on DCA treatment in CRC, we first determined miRNA expression profiles using a miRNA array (Figure 2A). In total, 119 miRNAs from 2059 mature human miRNAs were identified as candidate DCA-related microRNAs. Seven miRNAs (miR-340-3p, miR-124-5p, miR-877-3p, miR-487a-3p, miR-299-3p, miR-642b-5p and miR-543) were selected and further confirmed by quantitative real-time PCR (Figure 2B). The basal level of miR-543 in chemoresistant CRC cells was significantly higher than that in chemosensitive CRC cells (Figure 2C). Although several studies have revealed that miR-543 acts as an oncogene in various cancers 17-19, miR-543 has been reported to function as a tumor suppressor 20-22 . To validate the roles of miR-543 in the chemosensitivity of L-OHP, miR-543 was overexpressed in HCT116 and HCT-8 cells by directly transfecting cells with a miR-543 mimic or infecting cells with the overexpression virus. The IC50 of L-OHP in HCT116 and HCT-8 cells was increased in the miR-543 group (Figure 2D). In addition, miR-543 reduced the effects of L-OHP by increasing the levels of cleaved-PARP (c-PARP) and Bax, which are recognized biomarkers of cell apoptosis (Figure 2E). The colony formation associated with L-OHP treatment was significantly promoted by miR-543. (Figure 2F). These results suggest that miRNA-543 may play a crucial role in modulating chemosensitivity in CRC cells.

### MiR-543 negatively regulates PTEN and DCA upregulates PTEN through miR-543

**Figure 3:**
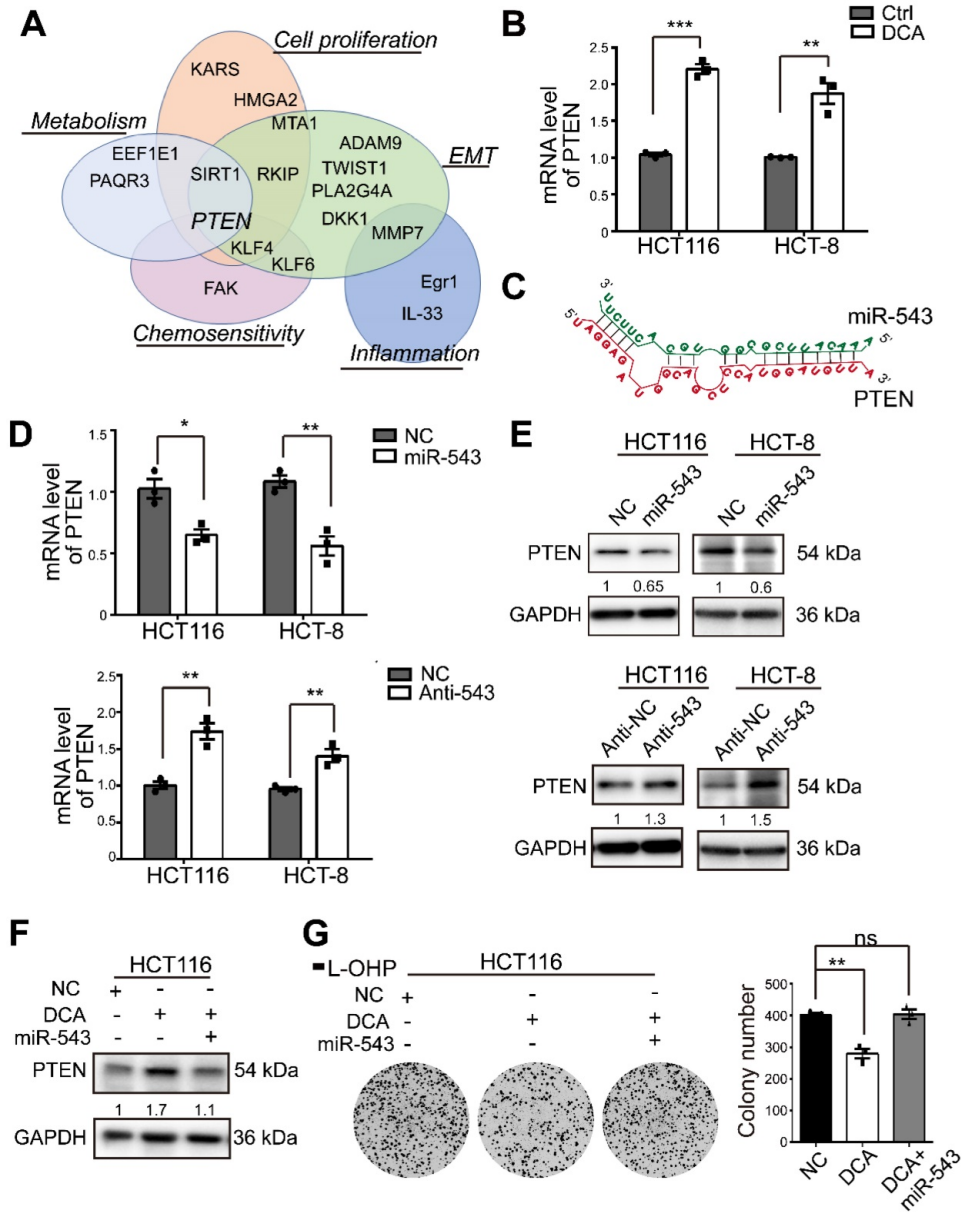
** PTEN is modulated by DCA/miR-543 in CRC cells.** (A) Examples of validated miR-543 targets and their biological functions. (B) The mRNA level of PTEN was determined by quantitative real-time PCR in HCT116 and HCT-8 cells treated with 20 mM and 15 mM DCA respectively for 24 hours. (C) Predicted structures of the potential binding site sequences of miR-543 with PTEN transcript by RNAhybrid. (D-E) HCT116 and HCT-8 cells were transiently transfected with miR-543 mimic or inhibitor. The mRNA and protein levels of PTEN were analysed. (F-G) HCT116 cells transfected with miR-543 mimic or NC were treated with or without DCA for 24 hours. The expression of PTEN and the colony formation assays were analysed. Representative results of three independent experiments are shown as the mean ± SEM, n = 3. *, *P* < .05; **,* P* < .01; ns, no significance.

To elucidate the mechanisms through which miR-543 exerts its regulation, several validated genes identified through a literature search were investigated (Figure 3A). Among these genes, PTEN, a key regulator of cell growth and apoptosis, has been reported to be involved in chemosensitivity^23^. The PTEN mRNA level was significantly upregulated by DCA (Figure 3B). The predicted binding sketch was obtained from RNAhybird (https://bibiserv.cebitec.uni-bielefeld.de/) (Figure 3C). To confirm a previous report, the miR-543 mimic or inhibitor was transfected into HCT116 and HCT-8 cells. PTEN mRNA expression was negatively regulated by miR-543 (Figure 3D). When miR-543 was overexpressed, the protein level of PTEN was significantly reduced in HCT 116 and HCT-8 cells. Moreover, abrogation of miR-543 resulted in elevated PTEN protein levels (Figure 3E). In addition, overexpression of miR-543 reversed the elevating effect of DCA on PTEN, indicating that DCA upregulates PTEN through miR-543 (Figure 3F). Meanwhile, overexpression of miR-543 abolished the inhibition effect of DCA in colony formation assay, which suggested that miR-543 plays a vital role in DCA enhancing chemosensitivity (Figure 3G).

### MiR-543 increases L-OHP chemoresistance* in vivo*

**Figure 4:**
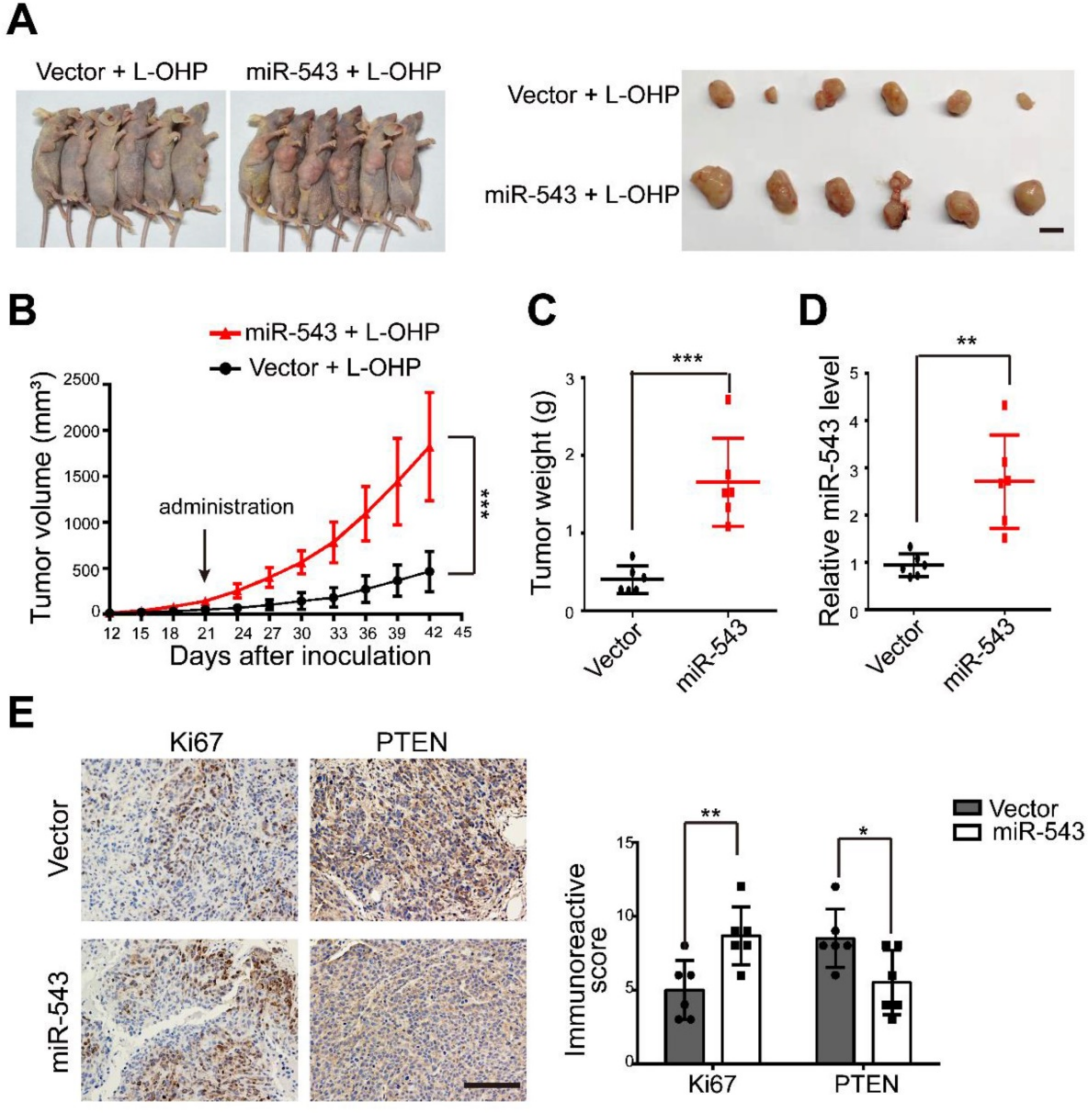
MiR-543 promoted L-OHP chemoresistance *in vivo.* (A) Representative images of six tumor-bearing mice in each group before sacrifice and representative images of dissected tumors in each group (Scale bars: 1 cm). (B) Xenograft tumor volumes were calculated for 42 days after tumor inoculation. (C) Tumor weights of the two groups were measured. (D) The expression of miR-543 was determined by quantitative real-time PCR in xenograft tumors. (E-F) Immunostaining of xenograft tumor sections with Ki67 and PTEN. Positive staining is indicated by the brown colour (Scale bars: 100 µm) (left panel). The immunoreactive score was calculated (right panel). The mean ± SD is shown *, *P* < .05; **, *P* < .01; ***, *P* < .001.

To evaluate the effect of miR-543 on increasing chemoresistance *in vivo*, control or stable miR-543-overexpressing HCT116 cells were subcutaneously injected into nude mice subcutaneously. Both groups were then intraperitoneally injected with L-OHP. Tumor growth was significantly promoted in the miR-543 group, as evidenced by images of tumor-bearing mice, tumor volume and weight (Figure 4A-4C). Successful overexpression ofmiR-543 was confirmed by real-time quantitative PCR (Figure 4D). The level of the cellular proliferative marker Ki67 was significantly increased in the miR-543 group. However, the expression of PTEN, as evidenced by immunohistochemical staining was reduced in the miR-543 group (Figure 4E). Thus, these results demonstrate that miR-543 promotes tumor growth with L-OHP treatment, and that PTEN was inhibited *in vivo*.

### MiR-543 activates the Akt/mTOR signaling pathway

**Figure 5:**
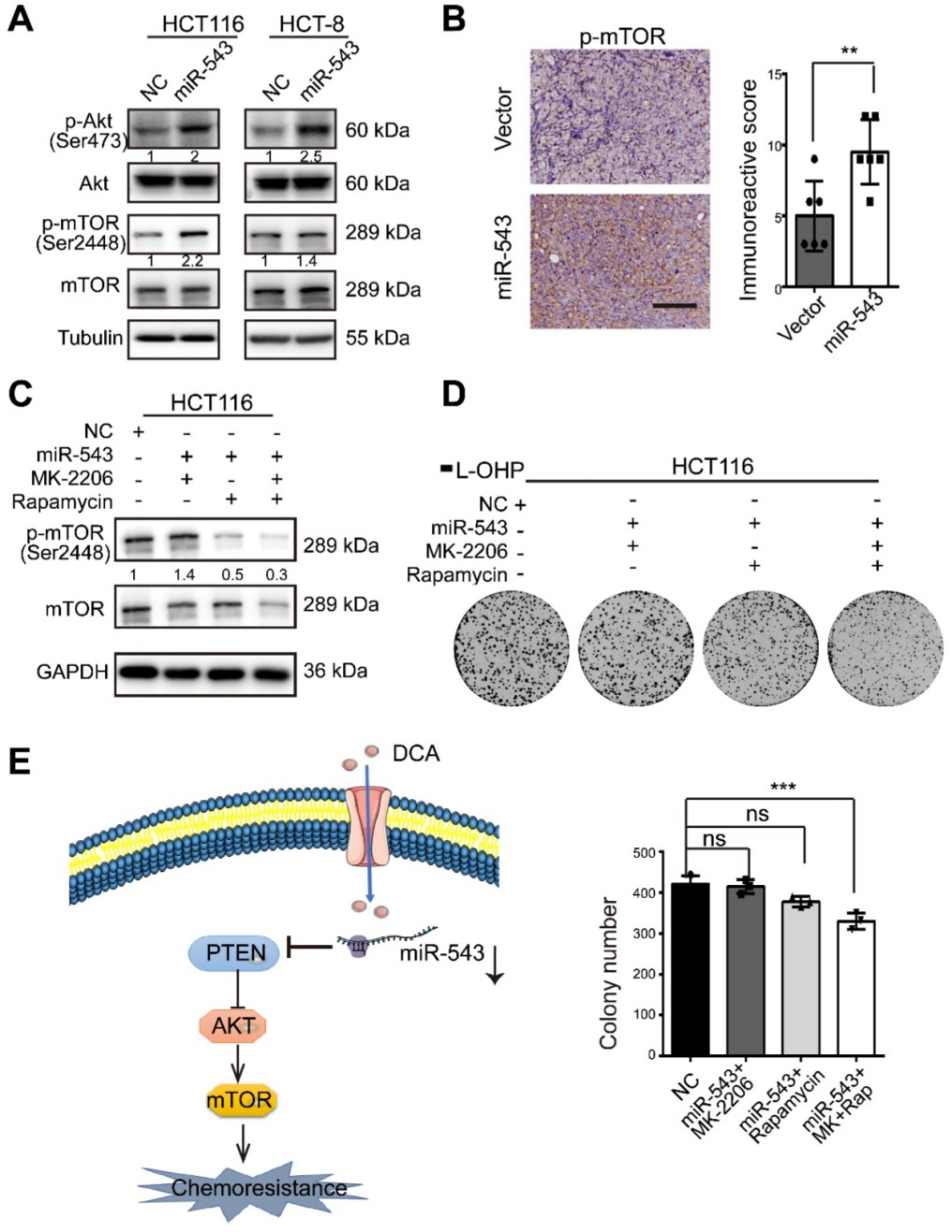
** Effects of miR-543 on the Akt/mTOR signaling pathways.** (A) The protein levels of p-Akt, Akt, p-mTOR and mTOR in HCT116 and HCT-8 cells transfected with miR-543 mimics were detected by Western blot analysis. (B) Xenograft tumor sections were immunohistochemically stained for p-mTOR. Positive staining is indicated by the brown colour (Scale bars: 100 µm) (left panel). The immunoreactive score was calculated (right panel). The mean ± SD is shown, n = 6; **, *P* < .01. (C-D) Overexpression of miR-543 in HCT116 cells in the presence MK-2206 and/or rapamycin. p-mTOR (Ser 2448) expression, mTOR expression and the colony formation assays were analysed. (E) A diagram model depicting the mechanism in which DCA overcomes chemoresistance through the miR-543/PTEN/Akt/mTOR pathway.

To further investigate if the Akt/ mammalian target of rapamycin (mTOR) pathway, a central pathway in cell proliferation, drug resistance and metabolism, is involved in the regulation of CRC by miR-543, the phosphorylation levels of Akt and mTOR was analysed. miR-543 promoted Akt phosphorylation and activated the downstream mTOR signaling pathway (Figure 5A). The subcutaneous tumor sections of mice shown in Figure 4 were stained, and the miR-543 overexpression group showed a higher level of p-mTOR (Figure 5B). These data suggest that the Akt/mTOR pathway is involved in the miR-543 induced chemoresistance. To assess this possibility, two small-molecule inhibitors, namely, MK2206 (AKT inhibitor) and rapamycin (m-TOR inhibitor), were used (Figure 5C). As shown in Figure 5D, both MK2206 and rapamycin inhibited the colony formation induced by miR-543, followed by inactivation of the mTOR pathway. These results suggest that the Akt/mTOR signaling pathway is involved in miR-543-mediated chemoresistance.

### MiR-543 expression is inversely correlated with PTEN in CRC patients

**Figure 6:**
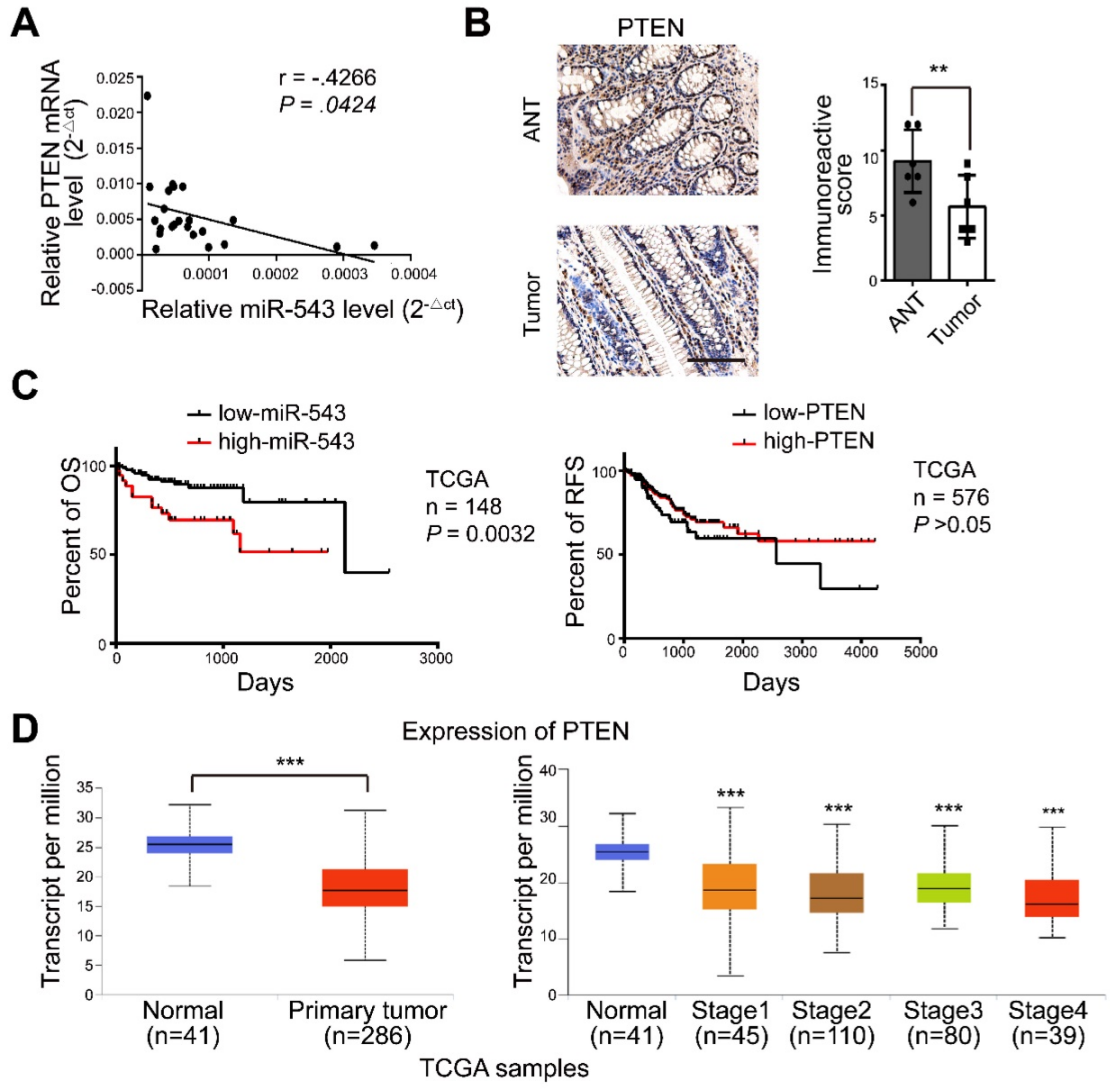
The expression of miR-543 is negatively related to PTEN in CRC patients. (A) The relationship between the expression of miR-543 and PTEN was determined using linear regression analysis in CRC patients. (B) Human tumor and normal tissues were immunohistochemically stained with PTEN. Positive staining is indicated by a brown colour (Scale bars: 100 µm) (left panel). The immunoreactive score was calculated (right panel). (C) The OS of CRC patients was stratified by miR-543 or PTEN expression in TCGA datasets (*P* values were obtained using the log-rank test) (D) The mRNA level of PTEN in CRC and normal tissues in TCGA samples.

We next explored these observations in human CRC tissues. There was a significant inverse correlation between miR-543 and PTEN mRNA levels in human CRC tissue (Figure 6A). The expression of PTEN in tumors and adjacent normal tissues (ANTs) was also detected via immunohistochemical staining. Figure 6B shows that the expression of PTEN in ANTs was significantly higher than that in tumor tissues. Furthermore, the survival analysis showed that CRC patients with high miR-543 expression were characterized by worse overall survival (OS) based on The Cancer Genome Atlas (TCGA) database but that no difference was observed in PTEN (Figure 6C). The expression of PTEN was also analysed in tumor tissue and normal tissue in TCGA CRC samples^24^, and the results showed that the mRNA level of PTEN was lower in the tumor tissue than in the normal tissue (Figure 6D). These results demonstrated that the expression of PTEN is negatively regulated by miR-543 in CRC patients and that higher miR-543 expression results in worse OS.

## Discussion

CRC is one of the leading causes of cancer mortality, and the development of chemotherapy resistance constitutes the main reason for treatment failure 2, 5. Here we reported that DCA restores the chemosensitivity of L-OHP resistant CRC cells through miR-543/PTEN/Akt/mTOR pathway.

As a structural analogue of pyruvate, DCA has been reported to inhibit PDK and convert pyruvate to acetyl-CoA, shifting energy generation from glycolysis to mitochondrial oxidative phosphorylation 25. Preclinical and clinical studies have revealed that DCA is a novel metabolic therapy for various cancer patients 26. Previous studies have shown that most experiments in cultured cell lines employ doses between 1 and 50 mM 25. The adverse effect of DCA in humans is generally limited to reversible sensory and motor peripheral neuropathy, which is influenced by age and genotype^27^. Previous studies have shown that DCA can overcome sorafenib and cisplatin resistance in hepatocellular carcinoma and head and neck cancer 11, 12. In addition, DCA induces cell apoptosis and cell cycle arrest in CRC cells 15, but DCA inhibits apoptosis during hypoxia 16. Thus, the effect of DCA on CRC cells, especially chemoresistant CRC cells remains elusive. The present study revealed that DCA sensitized chemoresistant CRC cells to L-OHP-induced cytotoxic effects and reduced colony formation via apoptosis.

PTEN has been characterized as a tumor suppressor in many human cancers^29^. The PTEN gene encodes a phosphatase protein that antagonizes the phosphoinositide 3-kinase (PI3K)/Akt/ mTOR antiapoptotic pathway. The PTEN/Akt/mTOR signaling pathway plays important roles in the transmission of proliferative signals, and this pathway may be aberrantly regulated in individuals with cancer and contribute to drug resistance and poor prognosis^30^. The present study revealed that the Akt/mTOR pathway is involved in the miR-543-induced chemoresistance, and miR-543 plays a supportive role in chemoresistance in CRC.

## Conclusion

In this study, we demonstrated that miR-543 promotes chemoresistance *in vitro* and *in vivo* and plays a vital role in DCA enhancing chemosensitivity. We found that DCA upregulates PTEN through miR-543, and disclosed that DCA restore chemosensitivity of L-OHP through the miR-543/PTEN/Akt/mTOR pathway.

## Figures and Tables

**Table 1:** Characteristic of CRC patients

Characteristic	Total(n=23)
Age-yr	65.1±9.4
Sex-no. (%)	
Male	13(56.5)
Female	10(43.5)
Localization-no. (%)	
Rectum	10(43.5)
Colon	13(56.5)
Staging- no. (%)	
T1 or T2	2(8.7)
T3 or T4	21(91.3)
Lymph nodes involved- no. (%)	
N0	8(34.8)
N1 or N2	15(65.2)
Metastasis- no. (%)	
M0	20(87.0)
M1	3(13.0)
Grade of differentiation- no. (%)	
G0 or G1	20(87.0)
G2 or G3	3(13.0)

**Table A:** The primer sequences.

ACTIN	F: 5'-CTCCATCCTGGCCTCGCTGT-3'
	R: 5'-GCTGTCACCTTCACCGTTCC-3'
PTEN	F: 5'- CAGAAAGACTTGAAGGCGTAT-3'
	R: 5'- TGGCGGTGTCATAATGTC-3'
miR-543	RT:5'-GTCGTATCCAGTGCGTGTCGTGGAGTCGG CAATTGCACTGGATACGACAAGAAGT-3'
	F: 5'- GGAAACATTCGCGGTGC-3'
	R: 5'- GTGCGTGTCGTGGAGTCG-3'
miR-642b-5p	RT: 5'- GTCGTATCCAGTGCGTGTCGTGGAGTCGG CAATTGCACTGGATACGACAGACACA-3'
	F: 5'- GGTTCCCTCTCCAAATG-3'
	R: 5'- CAGTGCGTGTCGTGGA-3'
miR-299-3p	RT: 5'- GTCGTATCCAGTGCGTGTCGTGGAGTCGGC AATTGCACTGGATACGACAAGCGGT-3'
	F: 5'- TATGTGGGATGGTAAAC-3'
	R: 5'- TGCGTGTCGTGGAGTC-3'
miR-340-3p	RT: 5'-GTCGTATCCAGTGCAGGGTCCGAGGTATTCG CACTGGATACGACGCTATA-3'
	F: 5'-GCGCGTCCGTCTCAGTTACTT-3'
	R: 5'-AGTGCAGGGTCCGAGGTATT-3'
miR-124-5p	RT: 5'-GTCGTATCCAGTGCAGGGTCCGAGGTATTCGCA CTGGATACGACATCAAG-3'
	F: 5'-CGCGTGTTCACAGCGGAC-3'
	R: 5'-AGTGCAGGGTCCGAGGTATT-3'
miR-487a-3p	RT: 5'- GTCGTATCCAGTGCGTGTCGTGGAGTCGGCAA TTGCACTGGATACGACAACTGGA-3'
	F: 5'- AATCATACAGGGACATC-3'
	R: 5'- TGCGTGTCGTGGAGTC-3'
miR-877-3p	RT: 5'- GTCGTATCCAGTGCGTGTCGTGGAGTCGGCAA TTGCACTGGATACGACCTGGGAG-3'
	F: 5'- TCCTCTTCTCCCTCCT-3'
	R: 5'- CAGTGCGTGTCGTGGAGT-3'
U6	F: GCTTCGGCAGCACATATACTAAAAT
	RT (R): CGCTTCACGAATTTGCGTGTCAT

**Table B:** The sequences of oligonucleotides

mimic-NC	5'-UUCUCCGAACGUGUCACGUTT-3'
mimic-543	5'- TGCGTGTCGTGGAGTC-3'
inhibitor-NC	5'-CAGUACUUUUGUGUAGUACAA-3'
inhibitor-543	5'-AAGAAGUGCACCGCGAAUGUUU-3'

## Abbreviations

CRC: colorectal cancer
DCA: Dichloroacetate
Oxaliplatin: L-OHP
HCT-8/L: L-OHP resistant HCT-8 cell
HCT116/L: L-OHP resistant HCT116 cell
miRNA: microRNA
